# Memory Retrieval-Extinction Combined With Virtual Reality Reducing Drug Craving for Methamphetamine: Study Protocol for a Randomized Controlled Trial

**DOI:** 10.3389/fpsyt.2020.00322

**Published:** 2020-04-29

**Authors:** Wang Liu, Xi-Jing Chen, Ya-Tong Wen, Markus H. Winkler, Pauli Paul, Yi-Ling He, Liang Wang, Hong-Xian Chen, Yong-Hui Li

**Affiliations:** ^1^Key Laboratory of Mental Health, Institute of Psychology, Chinese Academy of Sciences, Beijing, China; ^2^Department of Psychology, University of Chinese Academy of Sciences, Beijing, China; ^3^Department of Psychology I, Biological Psychology, Clinical Psychology, and Psychotherapy, University of Wurzburg, Wurzburg, Germany; ^4^Center for Mental Health, Women's Drug Rehabilitation Center of Guangdong Province, Foshan, China; ^5^Mental Health Institute, Second Xiangya Hospital, Central South Universit y, Changsha, China

**Keywords:** memory reconsolidation, retrieval-extinction, extinction, drug-associated memories, virtual reality, methamphetamine

## Abstract

**Background:**

Relapse, often precipitated by drug-associated cues that evoke craving, is a key problem in the treatment of methamphetamine use disorder (MUD). Drug-associated memories play a major role in the maintenance of relapse. Extinction training is a common method for decreasing drug craving by suppressing drug-associated memories. However, the effects are often not permanent, which is evident in form of spontaneous recovery or renewal of cue-elicited responses. Based on memory reconsolidation theory, the retrieval-extinction (R-E) paradigm may be more effective in decreasing spontaneous recovery or renewal responses than extinction. After the original memory reactivated to a labile state, extinction will be introduced within the reconsolidation window, thereby updating drug-associated memories. However, there are still some controversial results, which suggest that the reactivation of drug-associated memories and the 10 min-6 h of limited time window are two main elements in the R-E protocol. Virtual reality (VR) is supposed to promote memory reactivation by providing vivid drug-related stimuli when compared with movies.

**Objective:**

The aim of this study is to examine the effectiveness of R-E training combined with VR on reducing spontaneous recovery or renewal of cue-elicited responses, in comparison to extinction, R-E training provided outside the time window of 6 h and R-E training retrieved using videos, in methamphetamine abusers.

**Methods:**

The study is a parallel matched controlled study including 95 participants with MUD. Participants will be randomly assigned to either a R-10 min-E group (methamphetamine-related cues retrieval in VR followed by extinction after 10 min) or a NR-10 min-E group (neutral cues retrieval in VR followed by extinction after 10 min) or a R-6 h-E group (methamphetamine-related cues retrieval in VR followed by extinction after 6 h) or a RV-10 min-E group (methamphetamine-related cues retrieval in videos followed by extinction after 10 min). Cue-evoked craving and reactivity will be assessed at pre-test and at 1 day, 1 week, 1 month, and 6-month post-tests.

**Discussion:**

To our knowledge, this study will probably be the first study to examine the efficacy of R-E training combined with VR to reduce cue-evoked responses in people with MUD. This innovative non-pharmacological intervention targeting drug-associated memories may provide significant clinical implications for reducing relapse, providing the study confirms its efficacy.

**Clinical Trial Registration:**

The trial is registered with Chinese Clinical Trial Registry at 17 October 2018, number: ChiCTR1800018899, URL: http://www.chictr.org.cn/showproj.aspx?proj=30854

## Introduction

Methamphetamine is the commonly abused illegal drug recent years in China and entails great personal and societal costs (1, 2). Even after long periods of abstinence from drugs, the risk of relapse remains high in people with methamphetamine use disorder (MUD) (3, 4). Relapse is a core characteristic of substance use disorders (SUDs) and a major obstacle to successful treatment. Craving or cue reactivity elicited by drug-associated stimuli is invoked as a main motivating force behind relapse (5, 6). As drug-paired stimuli (cues, contexts, and behaviors) that are repeatedly associated with the reinforcing properties of drugs over the course of drug use, when subsequently encountered, are known to evoke craving or cue reactivity and then result in compulsive drug taking (5–7). Drug-associated memories supposed to be a primary trigger of drug craving and relapse. This suggests that effective treatments focused on the manipulations of the cue-drug memory to reduce cue-elicited craving or reactivity are needed for relapse prevention of MUD.

Extinction training is a common method used to decrease craving and reactivity evoked by drug-associated stimuli in an effort to reduce relapse propensity through suppressing the cue-drug memory (8). Initially, it had been assumed that repeated, unreinforced presentation of drug-associated stimuli (without drug administration), would “extinguish” cue-elicited craving and reactivity. Extinction training has been applied in a variety of forms to treat SUDs in clinical studies with varying levels of success (9). However, these cue-evoked responses frequently reemerge after the passage of time (spontaneous recovery) or in the presence of drug-associated stimuli different from the ones used in extinction training (renewal) (10, 11). This suggests that extinction might involve a new “cue-no drug” learning to inhibit or interfere with the initial “cue-drug” association instead of erasing the original memory trace (12, 13).

Recent studies have proposed that retrieval-extinction (R-E) training, based on the theory of memory reconsolidation, may be more effective in reducing spontaneous recovery or renewal of cue-elicited responses than extinction through modifying drug-associated memories (14, 15). Memory reconsolidation is a process to maintain and strengthen consolidated memories over time, during which previously consolidated memories re-stabilize after it is retrieved or “reactivated” (16). The R-E training follows the rationale to reactivate original drug-associated memories to a labile state by a brief and/or weak exposure to the drug-associated stimuli, and then extinction training will be used to interrupt the reconsolidation process of drug-associated memories within a limited time window by incorporating new information, thereby updating the original drug-associated memories (16). The 10 min-6 h of reconsolidation window has been examined in preclinical studies (17, 18) without enough clinical studies in SUDs. To our knowledge, only two translational studies have used R-E training to treat SUDs, which found that R-E training in 10 min-6 h, had better intervention effects on inhibiting spontaneous recovery (19) or renewal (20) of cue-elicited responses than extinction training. Nonetheless, other studies failed to replicate the results (21, 22). One of the key reasons is that the consolidated memories had not been reactivated to a labile state (23) for memory reconsolidation.

Virtual reality (VR), which has high ecological validity, may improve the effects of R-E training from a methodological perspective. VR can provide a variety of vivid drug-associated cues and contexts for individuals to interact with and the individuals with SUDs will be immersed in customized scenes by putting on a headset. Previous studies found that using VR to present drug-associated stimuli and interact with these stimuli during the retrieval process evoke craving more robustly than using traditional methods, such as pictures (24) and videos (25, 26). It suggests that VR may be a promising way to reactivate drug-associated memories by providing vivid drug-associated stimuli (25). Thus, combined VR with R-E training may be a prospective approach to treat MUD.

The primary objective of the present study is to examine the effectiveness of R-E training combined with VR in decreasing cue-elicited craving and reactivity in individuals with MUD, when compared to extinction training or R-E training provided outside the time window of 6 h. The second objective is to examine the effects of VR in promoting the reactivation of drug-associated memories during R-E training. The R-E training combined with VR will be compared with the R-E training combined with videos during the retrieval in decreasing cue-elicited responses. The third objective is to examine the effectiveness of R-E training combined with VR in attenuating spontaneous recovery and renewal of cue-elicited responses when compared to the other three interventions. All the cue-evoked craving and reactivity will be assessed at pre-test and 1 day, 1 week, 1 month, and 6-month posttests after intervention to investigate how long the effectiveness of R-E training will last. A novel methamphetamine-related scene will be added to the follow-up post-tests to assess if the effectiveness can translate to the new drug cue-induced craving and reactivity.

## Materials and Methods

### Design

The study will be a randomized controlled comparative clinical trial with two successive days of therapy and 6 months of follow-up. It will involve four parallel groups, namely a R-10 min-E group (methamphetamine-related cues retrieval in VR group), a NR-10 min-E group (neutral cues retrieval in VR group), a R-6 h-E group (group that will receive extinction training outside the reconsolidation window of 6 h) and a RV-10 min-E group (methamphetamine-related cues retrieval in videos group).

### Participants

This study will be conducted at the Changsha drug rehabilitation center, Changsha, Hunan, and Women' drug rehabilitation Center of Guangdong province, Foshan, Guangdong. The inclusion criteria are as follows: 1) age: 18–45 years old; 2) a history of using methamphetamine meets Diagnostic and Statistical Manual of Mental Disorders Fifth Edition (DSM-5) criteria for MUD (27); 3) abstinence periods at least 2 weeks without obvious withdrawal symptoms (e.g., drowsiness and dysphoria); 4) able to speak and read Chinese; 5) a signed consent form; and 6) cue-elicited responses including self-report craving, heart rate, skin conductance responses, and electroencephalogram power spectrum are all more obvious in methamphetamine-associated scenes than in neutral scenes in VR during the pre-test (28).

The exclusion criteria are as follows: 1) a history of using illicit drugs other than methamphetamine or methamphetamine tablets (e.g., heroin, cocaine, marijuana); 2) uncontrolled medical illnesses or psychosis; 3) use of any medication or medical condition that may affect cardiovascular function and mental state; 4) some kinds of nervous system diseases may influence performing the experiment (e.g., epilepsy, parkinsonism); 5) a history of head trauma that caused a coma lasting more than 30 min; 6) movement disorders; 7) hearing impairments; 8) color blindness or color amblyopia; 9) a vision or corrected visual acuity less than 1.0. The subjects with psychiatric comorbidities and other kinds of SUDs will be excluded using DSM-5 (27, 29).

### Study Procedure

Psychotherapists in the rehabilitation center will make the advertisements and announcement for recruitment. The participants will be screened by a psychologist for eligibility. Then, the researcher will meet the eligible participants, describe the study procedure, discuss with the participants on the questions they concerned, and ask each eligible participant for informed consent.

Firstly, participants will attend a 1 h individual face-to-face interview to collect demographic information, the use history (dosage, duration, frequency) of methamphetamine, cigarettes, and alcohol and the questionnaire on anxiety. The use history of methamphetamine, cigarettes, and alcohol and the anxiety index will be regarded as variables for the data analysis. Then, a VR using practice with a neutral scene lasting 6 min for accommodation will be implemented. Participants will adapt to VR scenes and know how to operate wireless controllers of the VR system. After this, participants will attend a VR cue reactivity assessment as a pre-test, during which participants will engage in two VR sessions (composed of a methamphetamine-VR scene for 3 min and a placebo-VR scene for 3 min) with a real-time recording of psychophysiological reactivity (heart rate, skin conductance reactivity, and electroencephalic response). There will be a 1 min break between the two sessions, and the placebo-VR scene will always be presented before the methamphetamine-VR scene to avoid the disturbance elicited by the methamphetamine-related cues. Self-report craving to methamphetamine-related cues will be rated after exploring both VR scenes. The pre-test will be used not only for measuring the baseline of cue-elicited responses but also for screening the participants who will respond more robustly to methamphetamine-associated cues in VR. According to the inclusion criterion, participants the cue-elicited responses including self-report craving, heart rate, skin conductance responses, and electroencephalogram power spectrum are all more obvious in methamphetamine-associated scenes than in neutral scenes during the pre-test will be included in groups (28).

Thereafter, participants will be randomly assigned to one of four therapeutic groups using a randomization table generated by a sequence generator in a computer for matching. One group (R-10 min-E group) will receive R-E training, namely memory retrieval of methamphetamine-related cues in VR followed by extinction training after 10 min. Another group (NR-10 min-E group) will receive extinction intervention, namely memory retrieval of neutral cues in VR followed by extinction training after 10 min. The other group (R-6 h-E group) will receive R-E training outside the time window of 6 h, namely memory retrieval of methamphetamine-related cues in VR followed by extinction training after 6 h. The RV-10 min-E group will receive memory retrieval of methamphetamine-related cues in videos followed by extinction training after 10 min. Psychophysiological reactivity will be recorded instantaneously during the two times of intervention. The cue-elicited responses during the retrieval session will be assessed to indicate memory reactivation (28).

**Figure 1:**
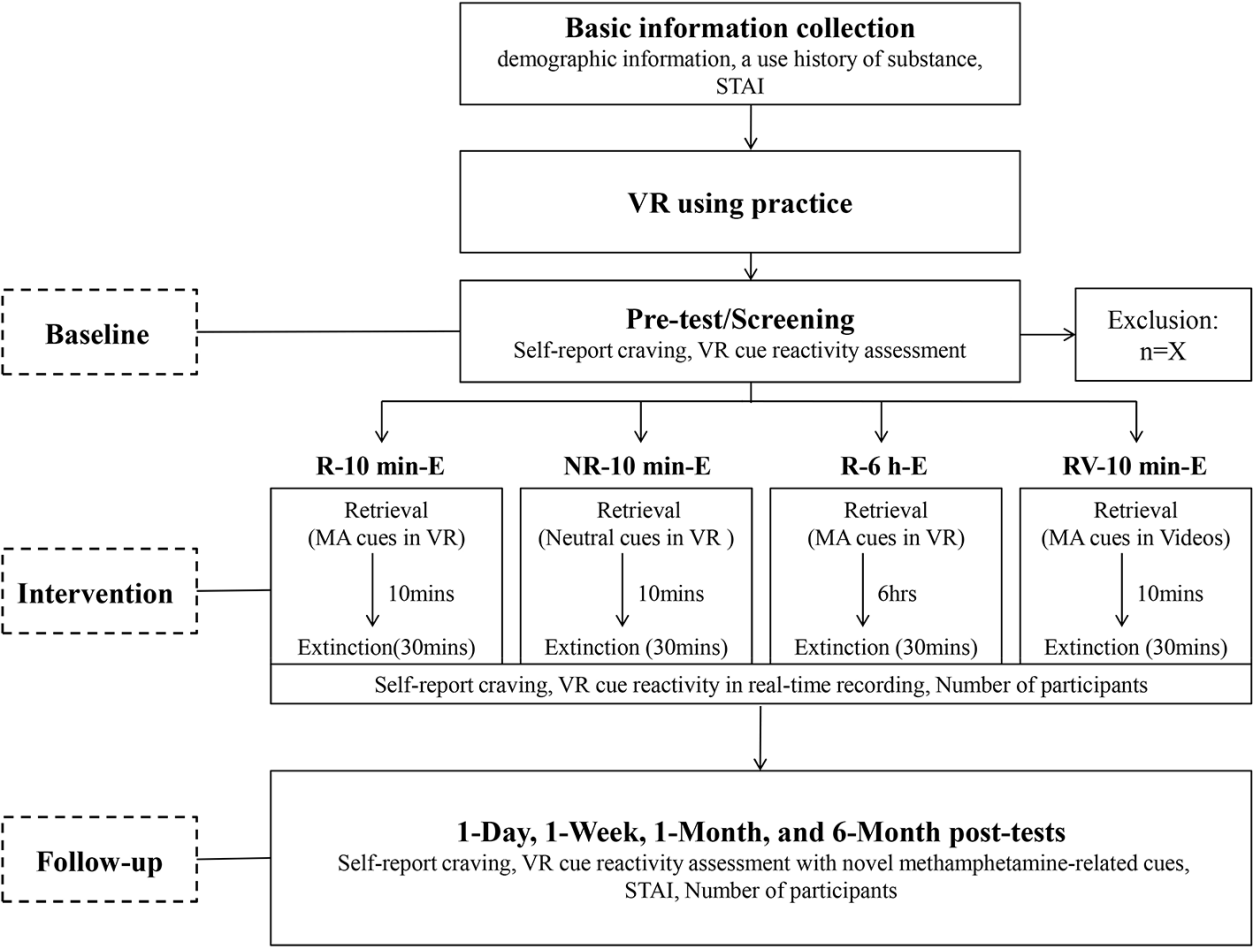
Flow chart of the study. STAI, State and Trait Anxiety Inventory; MA cues, Methamphetamine-related cues.

With the purpose of examining the effectiveness of R-E training in decreasing the spontaneous recovery of cue evoked responses, self-report craving and the VR cue reactivity assessment will be conducted at five different time points: pre-test, 1 day, 1 week, 1 month, and 6 months after the last intervention session. To measure the renewal effect, a new VR session with novel methamphetamine-related cues will be added to the VR cue reactivity assessment during the four post-tests compared to pre-test. Three psychotherapists with VR operation knowledge will conduct all the procedures for the four groups. The whole procedure of this study is depicted in a flow chart (**Figure 1**).

### Intervention

All four groups will receive two intervention sessions over two consecutive days (19, 20). The intervention process will be conducted by one of three psychotherapists including a 3 min memory retrieval session and a 30 min extinction training session. Psychotherapists should have technical ability to operate VR (self-developed through the collaborative work of the psychotherapists and researchers) to ensure the treatment's consistency and fidelity. The content of the extinction training session will be the same for the four groups. However, the intervention processes of four groups will differ in the content of the memory retrieval session and the time point of implementing extinction training.

#### R-10 min-E Group

Participants will receive two intervention sessions for the same, which both consist of memory retrieval for 3 min and extinction training for 30 min. The extinction training session will be implemented 10 min after the memory retrieval session. During the retrieval session, participants will be guided to explore a VR scene, which is considered as a high-risk situation for relapse, with both distal and proximal methamphetamine-related cues (e.g., a substance that appears to be methamphetamine, the water filter bottle of methamphetamine, straws, foil paper). In this way, the methamphetamine-associated memories of participants may be reactivated to a labile state.

During the extinction training session, six VR scenes with different methamphetamine-related cues in a room will be given to participants in six consecutive sessions. Participants will be asked to sit in front of a table on a sofa (or on a chair) in VR scenes. All the while, they will sit on a real sofa (or on a real chair) in the experimental environment in reality. Each VR session with one VR scene will last for 5 min. Six VR scenes will be presented in a randomized order to regulate individual differences in the relevance of craving-specific among participants. During the first and last minute of each VR session, participants will be instructed to observe the entire VR scene, and in the middle three minutes, a task for fully exposing methamphetamine-related cues will be assigned to the participants. After each session, participants are going to rate their craving to methamphetamine-related cues using a visual analog scale. Throughout the intervention process, psychophysiological cue reactivity will be recorded in real time.

#### NR-10 min-E Group

Participants will be provided with two intervention sessions for two days, both including 3 min of neutral cues retrieval followed by 30 min of extinction training 10 min after. The implementation of the retrieval session will guide participants to explore a neutral VR scene without methamphetamine-related cues. To ensure that the methamphetamine-associated memories of the participants will not be reactivated to a labile state, this session should be irrelevant to drug-associated stimuli.

The content of the extinction training session that the NR-10 min-E group will be exposed to is the same as the content that the other three groups will be exposed to.

#### R-6 h-E Group

Participants will receive two intervention sessions that the content of the retrieval session and the extinction training session will be identical to the R-10 min-E group. The only difference is that extinction training will be conducted 6 h after memory retrieval, when methamphetamine-associated memories are supposed to be reconsolidated again.

#### RV-10 min-E Group

Participants will receive two intervention sessions for two successive days. The duration and the time interval to implement the intervention sessions of the RV-10 min-E group are the same with the R-10 min-E group and the NR-10 min-E group. However, the method to present methamphetamine-associated cues of the RV-10 min-E group in the retrieval session is different from the other three groups. The participants will watch a video related to methamphetamine lasts 3 min to retrieve drug-associated memories. 10 min after the retrieval session, a same extinction training session will be implemented.

### Apparatus

The experiment is going to use HTC VIVE virtual reality system containing a headset (110 degrees, 1080×1200, 90Hz), two wireless controllers, and two base stations. Through the head tracker, participants can visually explore the VR scenes and walk around freely in a 3.0 m× 4.0 m space. The head orientation of participants will define the direction of locomotion. The wireless controllers will be used to interact with VR scenes and to provide their ratings on the visual analog scales. The VR scenes will be generated and run on a desk computer (Alienware 15-R2748, i7-7700HQ 16G 256GSSD+1T GTX1070 8G discrete graphics FHD). The required software is Microsoft Windows 10 (64-bit edition). The heart rate and skin conductance reactivity will be recorded by Biopac 16 Physiological multichannel instrument (BIOPAC MP150) including two transmitters and a signal projector connecting to a laptop. The electro encephalic response monitor has been developed by XinSi company in Beijing, China. The monitor has proven to be effective in recording electroencephalic responses (30).

### VR Scenes and Videos

The main software exploited to create the VR scenes is unity 3D. Unity is an ultimate and available game development platform used to build and deploy high-quality 3D games across VR. The neutral scene in the process of VR using practice will be a room with a desk, two spheres, two cubes, and a visual analogue scale. In a previous investigation of 60 people with MUD, four completely different VR scenes were constructed by unity 3D, two of which contexts were related to methamphetamine, while the other two were neutral contexts with neutral goods rather than methamphetamine-related cues. Two methamphetamine-related scenes are a living room and a bedroom with methamphetamine-related cues on a table or desk. To resemble the most familiar methamphetamine-related environment of participants in real life, a suitable VR scene will be chosen for each participant from the two methamphetamine-related rooms to be implemented in the pre-test, the memory retrieval session, and four post-tests (31). The other one of the two methamphetamine-related rooms which has not been chosen in the pre-test will be presented in four post-tests as a novel scene to test the renewal effect. Six methamphetamine-related scenes in the extinction training session are based on these two rooms with a double or a triple number of methamphetamine-related cues in different places of the rooms. One of the two neutral VR scenes has been prepared for the memory retrieval session that the NR-10 min-E group will view. The other one will be implemented in cue reactivity assessments as a baseline for the pre-test and post-tests to compare with methamphetamine-related VR scenes.

The dynamic VR scenes also provide participants with direct, realistic interactions, such as the grabbing of objects and physical or mechanical reactions to the user's presence. The four VR scenes including two methamphetamine-related scenes and two neutral scenes will be validated by a small number of people with MUD (n = 10) using self-report craving. All the VR scenes will be run in the software named Steam, which is a game platform as well.

The methamphetamine-associated video used in the retrieval session of the RV-10 min-E group will include an actress using *in vivo* mock methamphetamine paraphernalia (e.g. glass pipe, mock syringe, medical tubing, and a small plastic bag containing a substance that appears to be methamphetamine) to make a water filter bottle for administrating methamphetamine. The video will be validated by the same group of people with MUD (n = 10) using self-report craving. The video will be run on the desk computer.

## Outcomes

The experimental design will be double blinded. The craving and VR cue reactivity will be recorded by three psychologists during the pre-test, two intervention sessions, as well as the post-tests at 1 day, 1 week, 1 month, and 6 months after the intervention.

### Primary Outcomes

1. Self-report craving for methamphetamine will be measured using a 100-point visual analog scale in VR ranging from 0 (no craving) to 100 (high craving).
2. Psychophysiological recordings (see below for details) will be carried out at every VR cue exposure including two intervention sessions and VR cue reactivity assessments at the pre-test and the 1 day, 1 week, 1 month, and 6-month follow-ups.

### Secondary Outcomes

1. Anxiety will be measured with State-Trait Anxiety Inventory (STAI) (32). STAI is a widely used scale for general anxiety (33, 34). Chronic methamphetamine use may cause emotional dysfunction including anxiety (29). The Chinese version of STAI is a 20-item self-report instrument with a satisfying internal consistency (Cronbach's alpha = 0. 88) (35) and scores from 20 (absence of anxiety) to 80 (high anxiety).
2. Dropout rate will be assessed to indicate the motivation of participants to effectively engage in the treatment program (36, 37). Many methamphetamine users are reluctant to enter treatment and once in treatment there is an unacceptably high early dropout rate (38). Dropout rate is an important indicator may reflect the acceptability of the MUD treatments. The number of participants will be recorded at different time points for calculating the dropout rate.

### Psychophysiological Outcomes

1. Heart rate (HR) and heart rate variability (HRV) will be evaluated with the Biopac 16 Physiological multichannel instrument (BIOPAC MP150) during VR cue exposure at pre-test, two intervention sessions and four post-tests. HR reflecting the average heart rate is a kind of automatic response to emotional arousal. HR increases during anxiety states and decreases during relaxed states (39). As one of the main indicators of cue reactivity, the HR in individuals with SUDs may increase when they are exposed to drug-associated cues (5). The HRV indicates the fluctuations in HR around an average HR (30), which decreases during an anxious and exciting state and increases during a relaxed and calm state (39). HRV is also used as an autonomic index of emotion regulation capabilities (40), responding sensitively to drug-associated cues (41). HR and HRV are frequently regarded as objective measurements of anxiety responses and craving reactivity (5).
2. Skin conductance reactivity (SCR) will be recorded at the same time as the HR using BIOPAC MP150. Two Ag/AgCl electrodes of 20 mm×16 mm will be attached to the medial phalanges of the first and third fingers of the non-preferred hand of participants to obtain the signal of SCR. SCR is affected by emotional arousal, finger temperature, and finger activity. For recording SCR stably, participants will be told to keep the non-preferred hand down when they are exploring the VR scenes. SCR is considered to be an important index for cue reactivity evoked by drug-associated cues (30). During the state of craving, the blood vessels and sweat glands may change which will cause changes in the skin resistance, resulting in changes of the skin electricity. The spontaneous changes of SCR may be caused by R-E training, as demonstrated by a previous study (22). SCR has been used to measure craving in a previous methamphetamine study (42).
3. Electroencephalogram (EEG) will be recorded using an application of a mobile EEG equipment at the same time as the HR and the SCR. Four channels (TP9, FP1, FP3, TP10) will be kept to measure brain waves including alpha, beta, delta, and gamma frequencies. Previous studies on SUDs revealed that drug-associated cues evoked pronounced EEG power spectrum (43). The EEG has been used in previous studies as an objective index of cue reactivity to show craving (44).

## Sample Size

ANOVA was used to calculate the sample size (power = 80%, α = 5%). The calculation was based on the relevant data of a previous study of R-E training for individuals with tobacco use disorder, whose main results related to cue-elicited craving and reactivity (reference point =20%) (20). The result indicated that a minimum of 20 participants per group will be required for subsequent analysis. Taking into consideration that 15% of the participants may potentially drop out from the study, 95 eligible participants are needed for inclusion.

## Statistical Analysis

The means, medians, standard deviations, and ranges of the data will be summarized for quantitative data and counts and frequencies for categorical data. Primary outcomes and secondary outcomes will be analyzed separately. ANOVA (quantitative variables), Manna-Whitney-Wilcoxon test (ordinal variables), or Chi-square test (frequencies) will be used to compare outcomes between groups at baseline. Non-parametric tests will be used for data that are not normally distributed. Multiple imputations will be used to address the missing data if necessary. Furthermore, the mixed linear model analysis will be applied to compare outcomes among groups at follow-up assessments. Statistical significance is defined as p ≤ 0.05. Statistical analyses will be performed using SPSS statistics software, version 19.0 (SPSS Inc., Chicago, IL, USA).

## Discussion

To our knowledge, this study is the first intending to evaluate the effects of R-E training combined with VR on reducing methamphetamine-related craving and cue reactivity clinically. The primary findings of the randomized controlled trial may suggest that R-E training delivered by immersive VR may be highly effective for the reduction of craving and reactivity evoked by drug-associated stimuli in comparison to extinction and may ultimately decrease the rate of relapse. Another important point of the proposed study may be that using VR is more effective than using traditional methods to implement the memory retrieval during the reconsolidation intervention in decreasing cue-induced responses. This may provide the first proof that VR may improve the effects of the reactivation of drug-associated memories during R-E training.

First, this study pays close attention to MUD. In China, MUD constitute the majority of SUDs, especially among the youth (1, 2). Methamphetamine is a highly addictive psychostimulant drug that induces psychological dependence and has serious effects on mental health, posing a treatment challenge (45). Relapse is one of the main clinical problems in the treatment of SUDs (46), especially MUD, suggesting that more effective strategies are needed for relapse prevention in MUD.

Drug-associated memories may be the main factor of relapse in MUD. R-E training is expected to decrease cue-evoked craving and reactivity through a single reminder exposure to reactivate drug-associated memories prior to extinction training. A previous study showed that R-E training decreased conditioned fear response which was stubborn in case of spontaneous recovery, renewal (17). Furthermore, the findings of animal and human laboratory studies on conditioned fear are consistent with the reconsolidation hypothesis (18, 47, 48). As far as we know, the clinical utility of R-E training in SUDs has been examined in only two previously published studies. One study was about heroin use disorder, which found a marked reduction in self-report craving 6 months after R-E training, when compared to extinction or R-E training provided outside the time window of 6 h (19). The other one, which concerned nicotine use disorder, showed that using R-E training reduced drug craving elicited by novel drug-associated cues (20). These studies revealed significant clinical benefits of R-E training for inhibiting spontaneous recovery or renewal of cue-evoked craving and reactivity when compared to control groups. The consistent outcomes support the notion that the reactivation of drug-associated memories may be essential for disrupting the reconsolidation of original drug-associated memories by incorporating new information (extinction training) (16). Thus, the present study shares the same opinion with Xue et al. and Germeroth et al. on R-E training reducing spontaneous recovery and renewal of drug craving in SUDs. The effects of R-10 min-E training, NR-10 min-E training, and R-6 h-E training will be compared at 1 day, 1 week, 1 month, and 6-month follow-ups to test for the spontaneous recovery effect after the two intervention sessions, in the meanwhile, craving and reactivity elicited by novel methamphetamine-related cues will be assessed to test the renewal effect. Translational researches on R-E training may help people better understand the mechanism and process of memory reconsolidation. Also, clinical studies provide a new perspective on the treatment of SUDs (49).

Yet, there are still several inconsistent results about the effects of R-E training in some memory studies on conditioned fear (21, 22, 50, 51) or SUDs (52, 53), that may be due to the methodological differences (54) and inter individual differences between studies (55–57), which may result in limited reactivation of previously consolidated memories. From a theoretical perspective, VR appears to be a more useful method to reactivate drug-associated memories as drug-associated cues in VR elicit more stronger craving than drug-associated cues in pictures (24) or videos (25, 26). Using VR can not only present drug-related paraphernalia in proximal confrontation patterns, but also provide interaction with specific drug-related environment or multi-sensorial stimuli (distal risks), which are known to be the most critical triggers of relapse (58, 59). VR might thus offer high-risk methamphetamine-related environments. Then, the methamphetamine-related memories of participants may be reactivated to a labile, modifiable state more probably. To test this inference, the intervention effectiveness of the R-10 min-E group and the RV-10 min-E group in reducing cue-elicited responses will be compared in the present study. VR may be examined to promote the reactivation of drug-associated memories providing the intervention effectiveness is better through using VR to present drug-associated cues than using videos in the retrieval session. On the other hand, VR combined with extinction training may mitigate methamphetamine-related craving or extinguish cue reactivity. It is consistent with a previous study that VR combined with cue exposure treatment made progress in treating nicotine use disorder (60). Through this novel method, the relapse of people with MUD would be in good control when facing similar environments in reality.

There are several limitations to this study protocol. First, considering the feasibility and applicability of the study, the duration of exploring VR may cause discomfort due to the weight of the headset. Second, although VR scenes have been validated to be almost the same as the environments in which participants usually use methamphetamine, it is possible that a proportion of the participants may be unfamiliar with these situations, and therefore they may respond to new stimuli other than methamphetamine-related cues. Third, social interactions (with avatars in VR) are not included in these VR scenes. Future researches should consider employing more diverse designs involving social and personal drug-associated cues or triggers for dynamic plots (e.g., striking a light for methamphetamine or producing smoke) for VR scenes. In addition, there is no exact index to measure the extent of reactivation for drug-associated memories objectively. In this study or future studies, some psychophysiological measures combined with subjectivity experience may be regarded as a reference for the extent of reactivation. More important, as the participants will maintain the abstinence status in the rehabilitation centers when the study protocol are implemented, they will have no access to using methamphetamine that will make some objective measurements to test relapse rate infeasible, such as the urine test and hair test. These tests may be implemented after the participants get out of the rehabilitation centers to confirm the intervention effectiveness in the future.

The present study may extend the efficacy of R-E training on drug-associated memories by combining VR with R-E training to decrease cue-elicited craving and reactivity in people with MUD. The findings of the study may provide initial, compelling evidence that a brief R-E training in VR can attenuate methamphetamine-related craving and cue reactivity, which will have significant implications for relapse prevention and future studies on memory reconsolidation. VR will potentially become a maneuverable and low-cost approach for presenting controlled, individualized, and ecologically valid high-risk situations to people with SUDs receiving treatments. Ultimately, R-E training combined with VR may be a promising treatment for people with SUDs to prevent relapse.

## Ethics Statement

The ethics committee of the Institute of Psychology (CAS) has approved this protocol (H17015) and the study will be carried out in accordance with the recommendations of this committee. All participants will sign an informed consent form, providing they wish to do so, in accordance with the Declaration of Helsinki and with national and local regulations. The study is registered in the Chinese Clinical Trial Registry (www.chictr.org.cn) with the international standard randomized controlled trial number (ChiCTR1800018899).

## Author Contributions

WL designed and performed the experiments, and drafted the manuscript. X-JC and Y-TW prepared the published works and participated in the paper writing. MW and PP reviewed and edited the manuscript. LW, Y-LH and H-XC provided instructions for the study materials, computing resources, and laboratory instrumentation. H-XC helped to perform the experiments and to screen the participants. Y-HL guided the study design and directed the experiment implementation.

## Funding

This work was supported by the National Key Research and Development Program of China (2017YFC1310405), the Beijing Municipal Science and Technology Commission (Z171100000117014), the National Natural Science Foundation of China (U1736124), and the CAS Key Lab of Mental Health, Institute of Psychology.

## Conflict of Interest

The authors declare that the research was conducted in the absence of any commercial or financial relationships that could be construed as a potential conflict of interest.

## Abbreviations

MUD, Methamphetamine use disorder; R-E, Retrieval-extinction; SUDs, Substance use disorders; VR, Virtual reality.
